# Protective and Overprotective Behaviors against COVID-19 Outbreak: Media Impact and Mediating Roles of Institutional Trust and Anxiety

**DOI:** 10.3390/ijerph20021368

**Published:** 2023-01-12

**Authors:** Yi Liu, Cong Liu

**Affiliations:** School of Media and Communication, Shanghai Jiao Tong University, Shanghai 200240, China

**Keywords:** anxiety, COVID-19, institutional trust, media use, overprotective behavior, protective behavior

## Abstract

This study aims to explore how pandemic-related media use relates to both protective and overprotective behaviors and to probe the underlying mechanisms. The data were collected online during the early outbreak of COVID-19 in China, and a total of 1118 valid cases, which covered the 30 provincial administrative divisions in mainland China, were collected. Results showed that official government media use was positively associated with protective behaviors and institutional trust was an important mediator. Commercial media use was also found to be positively associated with overprotective behavior, and anxiety mediated this relationship. Findings of this study suggested that different media sources could play completely different roles. Institutional trust in government institutions and medical care systems were equally critical in translating the media effect into public compliance with the preventive measures advocated by the relevant departments. Media outlets and practitioners should also be responsible in order to avoid causing unnecessary anxiety among the public so as to reduce irrational overprotective behaviors.

## 1. Introduction

### 1.1. Background

Over the past two and a half years, COVID-19 has spread globally and brought multiple crises to countries and humanity. Due to the evolving threat of infection at the very early stage of the outbreak in 2020, hundreds of countries or regions declared states of emergency, borders were widely closed, billions of people were quarantined and worked from home, traditional forms of social interaction became difficult, and most countries and regions urgently enacted prevention and control policies and measures [1,2,3]. Although the tracing of the COVID-19 virus is still underway, in early 2020, Wuhan, China was the first major city in the world known to be severely hit by this pandemic [4]. In the face of serious public health emergencies and the hospitalization rate and mortality rate caused by the first generation of the coronavirus [5], the government at that time lacked prevention experience to deal with the pandemic, the public lacked scientific understanding of the virus, and there was a strong sense of tension in the entire Chinese society. During that period, China successively put forward pandemic prevention and control requirements such as the lockdown of cities and communities, requiring wearing masks in public places, taking body temperature, checking real-name QR codes, and so on [6].

At the same time, prime time television news, the front pages of newspapers, the top searches of the portals, and social media were flooded with information and reports about the pandemic. Global media coverage of the COVID-19 outbreak in China also exploded. The “infodemic” caused irretrievable public panic and inappropriate preventive behaviors, known as excessive prevention or overprotective behavior [7]. Excessive prevention refers to unnecessary measures which are more likely to be psychological comforts rather than preventive ones [8]. Typical overprotective behaviors such as panic purchases, excessive handwashing, and accidental poisoning with household disinfectants happened frequently, and bodily injury and even death caused by overprotective behaviors during the pandemic were reported in various countries [9,10,11].

Research on the social and behavioral reactions of the public when COVID-19 first broke out will become a critical reference in similar global public health crises in the future. Specifically, in the face of a completely unknown public health crisis, whether the public is willing to abide by the pandemic prevention policies proposed by relevant departments and implement them into individual health-protective behaviors against the crisis or whether they take irrational and excessive protective actions are questions worthy of research and consideration. In this process, it is also worth exploring indepth whether media information dissemination plays a positive or negative role and what the difference is between its impact mechanism on health protective behaviors and overprotective behaviors.

### 1.2. Relationship between Pandemic-Related Media Use, Protective Behavior, and Overprotective Behavior

According to the uncertainty reduction theory and media dependency theory, individuals have an unusually high need for information and sense-making during a severe social disruption [12,13,14]. In a crisis full of uncertainty and risk, the public may increase their dependence on the media as a response to the needs for risk evaluation based on reliable information sources, especially under the isolation and other control measures during the pandemic of COVID-19 [15,16]. Information-seeking from a variety of sources could reduce the uncertainty caused by the pandemic and ease anxious feelings [17,18]. Consumption of news about COVID-19 can shape social perceptions of the crisis in forms of judgment or attitudes which in turn influence protective behaviors. While moderate health-protective behaviors were found to be beneficial to subjective well-being and effectively reduce depression and anxiety disorders, excessive or irrational protective behaviors might have a negative impact on people’s normal lives and destroy their subjective well-being [19,20].

It is important to better understand the role of mass media in shaping the public’s knowledge, attitudes, and behaviors related to COVID-19 [21]. During the pandemic, a large amount of pandemic-related media use was indicated to be a double-edged sword. Previous studies have found that media use can promote health behaviors but also lead to irrational heath behaviors to a certain extent. On the one hand, researchers found that COVID-19 information exposure was positively associated with preventive behaviors and positive attitudes towards prevention, including wearing masks, washing hands regularly, avoiding crowds, and keeping social distance [22,23,24,25]. Higher information exposure was also found to be associated with COVID-19 vaccination acceptability among parents of those under the age of 18 as well as among the elderly [26,27]. It was argued that effective communication of facts helps individuals develop appropriate risk evaluations and make adaptive health decisions [28]. Both the traditional media and new media were found to be able to facilitate positive attitudes toward social prevention and increase the awareness of self and environmental hygiene during the COVID-19 outbreak [29,30].

While media use promotes health behaviors during a crisis, it may also cause conspiracy fantasies or negative emotions to promote overprotective behaviors, causing negative health effects or even death. The media is considered a major channel for spreading conspiracy beliefs and misinformation on medical as well as other topics, causing an infodemic [31,32]. Researchers argued that the influence of the infodemic, with mixed accurate and inaccurate information, on the media could possibly cause more negative influence than that of the pandemic during the early outbreak of COVID-19 [33]. The infodemic was reported to be able to cause individuals’ overuse of healthcare services, panic purchases, incompliance with preventive measures, and irrational protective behaviors [28,34,35,36,37,38]. Certain pseudoscientific practices spread through the media, from endorsing the use of hydroxychloroquine to recommending disinfectants as potential treatments for the virus, and caused numerous tragedies due to overprotective behaviors [39,40,41]. Experts also suggested limiting the exposure to news about the pandemic so as to prevent obsessive and compulsive symptoms such as excessive hand washing during COVID-19 [42].

**H1.**  *Pandemic-related media use is positively related to protective behavior.*

**H2.**  *Pandemic-related media use is positively related to overprotective behavior.*

### 1.3. The Mediational Role of Institutional Trust between Pandemic-Related Media Use and Health-Related Behaviors

The media may not only influence health-protective behaviors directly but also indirectly by influencing institutional trust. Institutional trust was usually defined as the trust that people place in a system or institution such as a government, a political party, the police, the courts, non-governmental organizations, public or private organizations, and so on [43,44]. Institutional trust is essential for the smooth operations of all interactions between governmental institutions and citizens, especially in times of crisis [45]. First, the news media were reported to play an important role in determining institutional trust. Studies suggested that various mass media sources influence citizens’ trust in government as well as trust in the ruling party [46,47,48]. In the context of COVID-19, media can be utilized as valuable tools for public engagement and information distribution so as to enhance the public trust in institutional authority and scientific expertise [49]. In situations where the pandemic crisis was highly complex and people lacked relevant knowledge, mass media were the essential channel for disseminating government policies as well as medical prevention and control measures to the public, making news consumption play an important role in public trust in public health institutions [21,50,51].

Second, institutional trust is further related to people’s willingness to follow rules and guidelines and adopt preventive behaviors [52,53,54], both generally and specifically to the time of the COVID-19 pandemic. Researchers believe that governments need to be trusted by citizens in terms of their ability to end the pandemic as well as the new measures of pandemic control, including testing procedures, tracing programs, or implementation of mass vaccination, so that the citizens are willing to comply with the policies and take appropriate actions [55,56]. A study found that Canadians exhibit high levels of compliance with policies on mask usage and that trust in public health officials plays a critical role [57]. In addition to government trust, trust in professional institutions also affects health protective behaviors. Trust in professional institutions is as important as government trust in influencing the public’s acceptance of the protection measures in the COVID-19 pandemic. Researchers found that maintaining and strengthening trust in politics, trust in science, trust in the CDC (Centers for Disease Control), and even trust in the WHO might be central in overcoming the COVID-19 pandemic [58,59].

**H3.**  *Institutional trust mediates the relationship between pandemic-related media use and protective behavior.*

**H3a.**  *Pandemic-related media use is positively related to institutional trust.*

**H3b.**  *Institutional trust is positively related to protective behavior.*

The relationship between institutional trust and overprotective behavior is comparatively less clear. Researchers have argued that under situations where the COVID-19 pandemic crisis was highly complex and people lacked key knowledge about it, trust in the key institutions issuing guidance to the public would increase the degree to which people hold accurate information for making appropriate decisions [21,50,51]. On the other hand, evidence among the limited empirical studies suggested that a high level of government trust was positively related to overprotective behaviors but only among those with low levels of COVID-19 knowledge [60]. Based on these inconsistent findings, the following research question was proposed:

RQ1: Does institutional trust mediate the relationship between pandemic-related media use and overprotective behavior?

### 1.4. The Mediational Role of Anxiety between Pandemic-Related Media Use and Health-Related Behavior

A Lancet study found that the pandemic increased the burden of depressive and anxiety disorders in 204 countries and territories in 2020 [61]. While the COVID-19 pandemic was becoming a serious global issue, information about the shortage of protective resources, the collapse of medical resources, and the rapid increase in the number of confirmed cases and deaths once dominated the media and had a great impact on social sentiment. In addition, various media sources were constantly loading information about the possibility of the virus staying active on various inanimate surfaces and stressing the importance of hygienic measures in the prevention of contamination, which added to the worries of contamination and overprotective behaviors [62]. Previous studies reported significant psychological strain or distinct mental conditions even outside the directly affected community resulting from the pandemic-related media coverage, especially when it is about collective trauma [63]. According to the vicarious traumatization theory, the emerging media made their audiences vicariously exposed to the vivid textual and also visual materials about the trauma survivors’ experiences under the pandemic, which became a major source of negative emotions such as anxiety [64]. During the COVID-19 pandemic, high levels of health anxiety, stress, and other negative affects were becoming common after exposure to stressful disease-related media content [64,65,66,67].

Anxiety was found to influence people’s ability to make rational decisions and to impact their behavior, and this was especially so during the active phase of the pandemic outbreak when mental health needs may not have been placed at the forefront of public health [68]. Excessive anxiety in response to a pandemic threat can become maladaptive and lead to irrational behavioral responses to prevent infection [50]. Anxiety is also considered to be the source of contamination fear, which is associated with compulsive rituals such as repetitive hand washing, cleaning, and taking disproportionate measures to reduce exposure to perceived sources of contamination [69]. Overprotective behaviors may lead to exacerbated problems, including medical system overload and even personal risks [70].

**H4.**  *Anxiety mediates the relationship between pandemic-related media use and overprotective behavior.*

**H4a.**  *Pandemic-related media use is positively related to anxiety.*

**H4b.**  *Anxiety is positively related to overprotective behavior.*

There is minimal and inconsistent evidence demonstrating the relationship between anxiety and health-protective behavior. It was found that anxiety can motivate both desirable and undesirable behaviors during the COVID-19 pandemic outbreak among Thailand’s citizens [71]. A study in China found that anxiety was not related to any differences in the adoption of preventive measures in response to the COVID-19 pandemic [72]. Another study in Japan showed that anxiety symptoms were linked to the use of fewer protective behaviors [73]. Based on these inconsistent findings, the following research question was proposed:

RQ2: Does anxiety mediate the relationship between pandemic-related media use and protective behavior?

Gaps remain in the existing literature. First, it is believed that the media impact the protective and overprotective behaviors against COVID-19 as well as that the potential underlying mechanisms vary. An overarching study picturing such relationships is lacking, nor are there studies comparing the media effect on both protective and overprotective behaviors. Second, existing studies on media influence during the COVID-19 pandemic mainly focused on social media use, and a few of them inferred online news consumption generally. However, social media cover divergent media sources, from official government media and commercial media to enterprise and individual accounts and acquaintances. In the Chinese media context, official government media and commercial media as the major pandemic information sources are quite different genres with different contents of reports spreading on social media and other online platforms. It is barely known how different media sources influence health-related behaviors or institutional trust and anxiety. Third, most existing studies focus on governmental or political trust and its relationship with media use and compliance behaviors, while it is more meaningful to include this variable in the context of the COVID-19 pandemic. It was argued that healthcare professionals and politicians in particular have to take the concept of “institutional trust” seriously if they are to improve their commitment to public health [44,74]. Therefore, institutional trust incorporating trust in both the government departments and medical care system, which are the two critical institutions involved in the COVID-19 pandemic, should be more highlighted in relevant studies.

### 1.5. Research Conceptual Model

**Figure 1:**
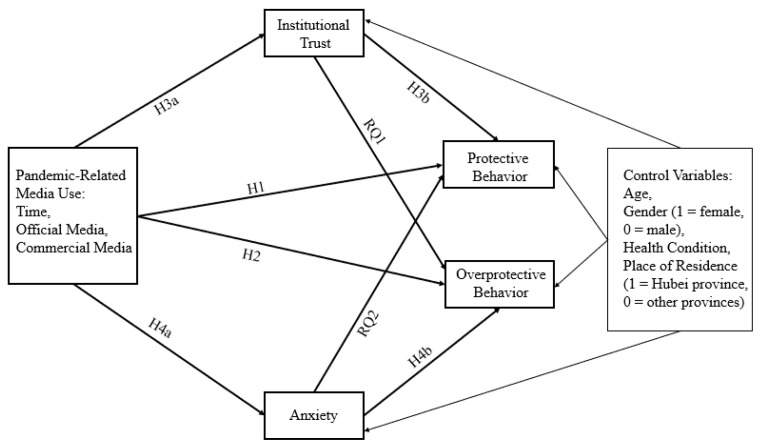
Conceptual framework.

To fill the gaps in the existing studies, this current study will explore how pandemic-related media use influenced both protective and overprotective behaviors during the early outbreak of COVID-19. Pandemic-related media use focuses on official government media use and commercial media use. In addition, the underlying mechanisms of the media impact will be further probed by analyzing the mediation roles of institutional trust (incorporating government trust and trust in medical care system) and anxiety. Based on the above hypotheses and research questions, we proposed the following conceptual model, controlling for the effect of age, gender, health condition, and place of residence (Figure 1).

## 2. Methods

### 2.1. Study Design

The data were collected online by a data collection service provider (i.e., Changsha Ranxing IT Ltd., Changsha, China) with more than 2.6 million members all over China. During the early outbreak of COVID-19 in China in September 2020, the company invited 5203 members from 30 provinces of mainland China via webpages and WeChat to participate in the online survey, and 1342 of them responded. A total of 1118 valid responses were retained, with a response rate of 21.5%. The final sample covered 30 provincial administrative divisions in mainland China, and it comprised the cities in Hubei province which were most severely influenced by the pandemic, six cities affected relatively severely by the pandemic (Beijing, Shanghai, Guangzhou, Shenzhen, Chongqing, and Wenzhou), and other cities affected less severely by the pandemic. Based on the meta-analysis results regarding the influence of social networking sites on health behavior change, a Hedge’s g of 0.24 was obtained, indicating small to medium effect sizes. According to [75], to obtain a power of 95% for a two-tailed alpha of 0.05, a sample size of 906 was required, and hence the sample size of 1118 of the current study is considered reasonable.

### 2.2. Participants

Among the participants, 45.9% were males and 54.1% were females; a majority of them were aged between 18 and 40, and about 80% of them had a university degree. Additionally, 24% reported an average health condition, 53.7% reported a good health condition, and 20.3% reported an excellent health condition. One-fifth (23%) of the participants lived in Hubei (4.7% of them lived in Wuhan), 36% were living in cities with severe effects of the pandemic at the time of data collection (including Beijing, Shanghai, Chongqing, Guangzhou in Guangdong province, Shenzhen in Guangdong province, and Wenzhou in Zhejiang province), and 41% were living in other cities. Characteristics of the participants are shown in Table 1.

### 2.3. Measurements

#### 2.3.1. Pandemic-Related Media Use

Pandemic-related media use measured participants’ time spent on pandemic information (1 = hardly ever; 5 = more than 5 h) and pandemic information acquisition from official government media (e.g., CCTV, People’s Daily, Hubei Daily, Changjiang Daily, etc.) and commercial media (e.g., Sanlian Life Week, Caixin, The Paper, Infzm, etc.; 1 = hardly ever; 5 = often).

#### 2.3.2. Protective Behavior

Participants were asked to rate their protective behaviors during the early outbreak of COVID-19. The measurement included four preventive actions that were suggested or required by the Chinese government: “I wear a mask when I go out”, “I increase the frequency of hand washing and regular disinfection”, “I try to minimize the opportunity of going out”, “I keep distance from others in public”. The responses were coded dichotomously (1 = yes; 0 = no). Cronbach’s alpha was 0.73 for this scale.

#### 2.3.3. Overprotective Behavior

Participants were asked to rate their overprotective behaviors during the early outbreak of COVID-19. The measurement included three items describing the commonly reported overprotective behaviors: excessive disinfection (“I excessively wash my hands and disinfect”), panic purchases (“I always overstock food and medicines”), and social withdrawal/phobia (“I always suspect that someone around me is a carrier of the coronavirus”). The responses were coded dichotomously (1 = yes; 0 = no). Cronbach’s alpha was 0.74 for this scale.

#### 2.3.4. Anxiety

The measurement of anxiety was adapted from Zung’s Anxiety Status Inventory (ASI) [76]. Participants were asked to rate their anxiety status during the early outbreak of COVID-19. It comprised three questions: “I feel nervous and anxious due to the pandemic”, “I have had times when I felt myself trembling and shaking during the pandemic”, and “I have sleeping problems during the pandemic”. The responses were rated on a 5-point scale (1 = totally disagree; 5 = totally agree). Cronbach’s alpha was 0.83 for this scale.

#### 2.3.5. Institutional Trust

Participants were asked to evaluate their institutional trust during the early outbreak of COVID-19. Ten items were adopted from previous studies measuring institutional trust, including both government trust and trust in the medical care system [77,78]. The descriptions included “I trust the advice of the medical staff”, “I trust the opinions and suggestions of the experts”, “I trust the findings of the medical researchers”, “I believe that the local health institutes can provide adequate medical services and have sufficient treatment capabilities”, “I am full of confidence in the treatment I will receive in case I get infected with COVID-19”, “I believe that the medical system will protect the privacy of patients”, “I support the government policy of wearing masks in public occasions”, “I support the lockdown policy in some cities and residential areas”, “I support the isolation policy of the close contacts of COVID-19 patients”, and “I support the policy of national epidemiological surveys including measuring body temperature, reporting travel records, etc.”. The responses were rated on a 5-point scale (1 = totally disagree; 5 = totally agree). Cronbach’s alpha was 0.73 for this scale.

### 2.4. Data Analysis Plan

Pearson correlation was first conducted to demonstrate the relationships among the key variables using SPSS. The hypotheses and research questions were then tested by conducting the structural equation modeling technique using AMOS.

## 3. Results

### 3.1. Correlation Analysis

Pearson correlation analysis (Table 2) showed that protective behavior was positively correlated to official government media use (r = 0.09, *p* < 0.01) and institutional trust (r = 0.29, *p* < 0.001) and was not significantly correlated to time spent on pandemic information (r = 0.06, *p* > 0.05), commercial media use (r = 0.01, *p* > 0.05), or anxiety (r = 0.06, *p* > 0.05). Overprotective behavior was positively correlated to time spent on pandemic information (r = 0.23, *p* < 0.001), commercial media use (r = 0.22, *p* < 0.001), and anxiety (r = 0.60, *p* < 0.001) and was not significantly correlated to official government media use (r = 0.06, *p* > 0.05) or institutional trust (r = 0.03, *p* > 0.05). There was a positive relationship between protective behavior and overprotective behavior (r = 0.10, *p* < 0.05).

### 3.2. Structural Equation Modeling Predicting Protective and Overprotective Behaviors

Confirmatory factor analysis (CFA) was first performed for testing the measurement model with three observed variables (i.e., time spent on pandemic information, official government media use, and commercial media use) and four latent variables (i.e., trust, anxiety, protective behavior, and overprotective behavior). The measurement model obtained good model fit (χ^2^ = 790.34, df = 209, CFI = 0.90, RMSEA = 0.05), and all the items significantly loaded to the assumed latent variables (*p* < 0.001), indicating robust measurements to the latent constructs.

**Figure 2:**
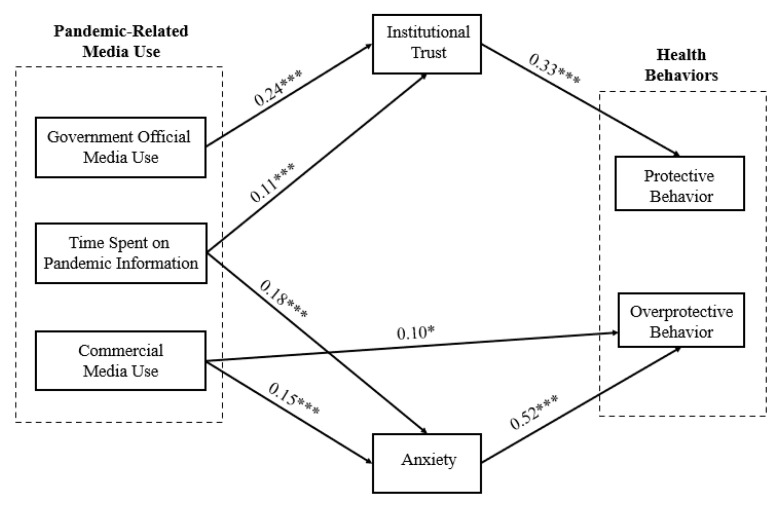
Structural equation modeling among pandemic-related media use, institutional trust, anxiety, protective behavior, and overprotective behavior (*** *p* < 0.001, * *p* < 0.05).

Second, a path analysis was established for testing the structural model among pandemic-related media use (including time spent on pandemic information, official government media use, and), institutional trust, anxiety, protective behavior, and overprotective behavior, controlling for the effect of gender (1 = female, 0 = male), age, health status, and place of residence (1 = Hubei province, 0 = other provinces). A good model fit was obtained (χ^2^ = 60.72, df = 16, CFI = 0.95, RMSEA = 0.05). Results (Figure 2) showed that time spent on pandemic information was positively correlated to institutional trust (β = 0.11, *p* < 0.001) and anxiety (β = 0.18, *p* < 0.001) but not to protective or overprotective behaviors (β = 0.01, *p* > 0.05; β = 0.08, *p* > 0.05). After controlling for the effect of time spent on pandemic information, official government media use was found to be positively related to institutional trust (β = 0.24, *p* < 0.001) but not to anxiety (β = −0.01, *p* > 0.05), protective behavior (β = 0.05, *p* > 0.05), or overprotective behavior (β = −0.01, *p* > 0.05); commercial media use was positively related to anxiety (β = 0.15, *p* < 0.001) and overprotective behavior (β = 0.10, *p* < 0.05) but not to institutional trust (β = −0.02, *p* > 0.05) and protective behavior (β = −0.03, *p* > 0.05). In addition, institutional trust was positively related to protective behavior (β = 0.33, *p* < 0.001) but not to overprotective behavior (β = −0.02, *p* > 0.05). Anxiety was positively related to overprotective behavior (β = 0.52, *p* < 0.001) but not to protective behavior (β = 0.05, *p* > 0.05). Among the control variables, age was negatively correlated to overprotective behavior (β = −0.13, *p* < 0.01). Health condition was positively correlated to institutional trust (β = 0.17, *p* < 0.001) and negatively correlated to anxiety (β = −0.17, *p* < 0.001) and overprotective behavior (β = −0.08, *p* < 0.05). Place of residence was positively correlated to overprotective behavior (β = 0.09, *p* < 0.05). All other links between the control variables and the four endogenous variables were nonsignificant.

Regardless of participants’ time spent on pandemic information, institutional trust significantly mediated the link between official government media use and protective behavior (β = 0.08, *p* < 0.05); meanwhile, anxiety mediated the link between commercial media use and overprotective behavior (β = 0.08, *p* < 0.05).

H1 was not supported as there was no direct correlation between any of the factors of the pandemic-related media use and protective behavior. H2 was partially supported and commercial media was directly correlated to overprotective behavior. H3 was partially supported and institutional trust was found to mediate the relationship of official government media use with protective behavior. H4 was partially supported and anxiety was found to mediate the relationship of commercial media use with overprotective behavior. The answers to RQ1 and RQ2 were negative.

## 4. Discussion

This study explored the impact of pandemic-related media use on protective and overprotective behaviors during the early outbreak of COVID-19 in China, with institutional trust and anxiety as the mediators. It was found that after controlling for time spent on pandemic information, official government media use was indirectly related to protective behavior, and institutional trust was an important mediator between them; meanwhile, there was not only a direct correlation between commercial media use and overprotective behavior but also an indirect association between them mediated by anxiety.

Generally, the findings suggested that media is a double-edged sword with both positive and negative impacts on health-related behaviors during a public health crisis, and different media sources play different roles. On the one hand, official government media use for pandemic information seeking was found to be positively linked to protective behaviors. In the face of an unknown pandemic crisis, people lacked relevant knowledge and coping experience and tended to be reliant on media information for risk assessment, including how the pandemic threatened them and how safe their living and working environments were [13]. In addition, the public also needs to rely on the media to obtain relevant and reliable information for pandemic prevention, such as the information about medical care, vaccination, and first-time official pandemic prevention policies. The official government media in China play the role of the spokesman of the government. The content released pays attention to the authority, seriousness, and objectivity. Specifically, government prevention and control agencies need to transmit important information to the public via official media in a timely manner, not only to inform the public of scientific preventive measures to urge the citizens to take self-protection measures but also to update of public policies for pandemic control in real time, such as use of real-name health codes and travel cards, nucleic-acid testing regulations, and other information. Therefore, official government media delivers scientific prevention information to the public, helping them form a scientific understanding of the virus and scientifically adopt the protective behaviors to improve immunity and prevent infection. In other words, the official government media play a critical role during the pandemic in encouraging the public to fight against the social crisis in an effective and healthy state. On the other hand, however, findings in this study also showed that commercial media use was positively linked to overprotective behaviors. Commercial media need to maintain and improve their market share from the exclusive perspectives of reports. In the early days of the COVID-19 outbreak, Chinese commercial media tended to record the unfortunate encounters and real traumas from a microscopic perspective, and the examples include “After the City Was Closed, My Sick Father and I Were Separated” and “A Teenager Who Grew Up Overnight in the Pandemic”. There were overwhelmingly a large number of similar reports released by commercial media which were disseminated quickly and widely. The flooded pandemic-related reports on commercial media are more likely to form an infodemic, which may trigger panic and vicarious trauma, exacerbate the public anxiety, and cause irrational or excessive protective behaviors [34,64,79].

Second, an insightful implication of this study is that institutional trust is an important factor translating the media effect into people’s health-promoting behaviors, especially for that of the official government media. In other words, media exposure to official government media could form a positive attitude toward relevant institutions, which further cultivates the public’s behavioral intentions to compliance or actual compliance behaviors. This is supported by previous findings which showed that people in high-trust regions decreased their non-necessary activities more often than those in low-trust regions in times of COVID-19, indicating that degraded trust and cohesion could have dramatic consequences when compliance is required for collective survival [80]. Similarly, researchers believed that public trust in the government’s capability to manage the pandemic is crucial as it underpins public attitudes and behaviors at this precarious time of public health [81]. Data from China also demonstrated that trust in local government and media helps to reduce the infection rate of COVID-19 [82]. Sufficient institutional trust facilitates the transversion from the media content to the compliance behaviors of the public, and this function of institutional trust is especially crucial for the dissemination of professional guidance from relevant institutions [83]. These findings suggest that maintaining a good institutional image reflected in the media is helpful in consolidating public trust and confidence in institutions, which in turn effectively facilitates the work of public persuasion, dissemination of key preventive information, and guidance for the public to implement a series of protective behaviors. In addition, in the highly mediated environment, maintaining the figures of public health officials should not be the only focus, and how healthcare professionals and experts behave in the media is also consequential for perceived institutional trustworthiness. Researchers suggest that scientists should show more openness, that is, the willingness to listen or to give others a voice [84].

Third, another important implication of the findings of this study is that media practitioners, especially commercial media, should take responsibility to avoid creating excessive anxiety and thus unnecessary irrational protective behaviors. Firstly, whether a topic is appropriate to occupy the media interface with high frequency is worth thorough consideration by media outlets as the presentation of high-frequency and multi-content news may cause information overload and vicarious trauma among the people. Secondly, from the perspective of media ethics, we call on all media to strengthen humanistic care for their audiences and avoid aggravating anxiety in society. Media platforms should be cautious about objectivity and authenticity, interviews, and the reporting of the story, and rational and scientific reports are more encouraged to avoid public anxiety. In the process of crisis news production, use of the conflict framework between humans and the coronavirus may not be the optimal way; instead, reports from perspectives of health communication and popular science communication are more beneficial in guiding the public to respond to the pandemic and protect themselves with a scientific attitude and with positive emotion.

This study has several limitations. First, although the participants covered 30 provinces in mainland China, the sample size of 1118 could have limited the representativeness of subgroups of different age, gender, income, and ethnicity. Second, since the data were collected online, the participants could be more educated, more internet active, and more urban than the general adult population in mainland China. Future studies should try to recruit a larger sample size and cover more participants from minority groups and suburb areas so as to better represent the entire Chinese population. Third, the COVID-19 infection rate was very low at the early stage of the outbreak, and only 0.2% of the participants were actually infected. It would be more meaningful to explore in future studies the situations among people who were infected. Fourth, the institutional trust measured in this study (e.g., “I support the lockdown policy in some cities and residential areas.”) implied discretionary behaviors, whereas in most places across the globe, community behaviors were not discretionary and people were required to behave according to tgovernment mandates. Fifth, it is inappropriate to make causal conclusions due to the cross-sectional nature of this study, and the results need to be interpreted with caution. Longitudinal studies tracking participants’ attitudinal and behavioral changes over time during the COVID-19 pandemic are more optimal for explaining the media impact on protective and overprotective behaviors.

## 5. Conclusions

With the firsthand data collected during the very early stage of the COVID-19 outbreak in Wuhan and other cities in China, the current study explored how pandemic-related media use relates to both the protective and overprotective behaviors against the completely unknown COVID-19 as well as the mediation roles of institutional trust and anxiety. The findings suggest that the media impact during the early outbreak of COVID-19 was a double-edged sword that related to both the protective and overprotective behaviors of the public. Specifically, more official government media use related to more protective behaviors, whereas more commercial media use related to more overprotective behaviors. Meanwhile, the underlying mechanisms varied. The positive media impact of official government media worked through institutional trust, and the negative media impact of commercial media worked through anxiety emotions. Institutional trust in government institutions as well as the medical care system could be equally critical in translating the media effect into the public compliance with the preventive measures advocated by the relevant departments. In addition, media outlets and practitioners should be responsible and avoid causing unnecessary anxiety among the public so as to avoid irrational overprotective behaviors.

## Figures and Tables

**Table 1:** Participant characteristics.

Demographics	Percentage	*n*
Gender		
Male	45.9%	513
Female	54.1%	604
Age		
<18	4.1%	46
18–25	30.6%	342
26–30	22.6%	252
31–35	23.5%	263
36–40	9%	101
41–50	7.6%	85
51–60	2.3%	26
>60	0.2%	2
Education		
Primary school	0.8%	9
Junior high	3.7%	41
High school	8.2%	92
College/university	79.8%	892
Master’s, doctoral degrees and above	7.5%	84
Health Condition		
Very poor	0.1%	1
Relatively poor	2.0%	22
Average	24.0%	268
Relatively good	53.6%	599
Very good	20.3%	227
Place of Residence		
Hubei province	23.0%	257
Other provinces	77.0%	860

*n* = 1118.

**Table 2:** Pearson correlation among the key variables.

	(1)	(2)	(3)	(4)	(5)	(6)	(7)
Time spent on pandemic information (1)	1	0.24 ***	0.23 ***	0.22 ***	0.17 ***	0.06	0.23 ***
Official government media use (2)		1	0.35 ***	0.07 *	0.26 ***	0.09 **	0.06
Commercial media use (3)			1	0.17 ***	0.10 **	0.01	0.22 ***
Anxiety (4)				1	−0.01	0.06	0.60 ***
Institutional trust (5)					1	0.29 ***	0.03
Protective behavior (6)						1	0.10 *
Overprotective behavior (7)							1

*** *p* < 0.001, ** *p* < 0.01, * *p* < 0.05.

## Data Availability

The data presented in this study are available on request from the corresponding author.
